# Supplementary material for: Distinct regulatory ribosomal ubiquitylation events are reversible and hierarchically organized
Source: eLife. 2020 Feb 3;9:e54023. doi: 10.7554/eLife.54023 (PMC7064338; doi:10.7554/eLife.54023)
Supplement: Supplementary file 1. [file elife-54023-supp1.docx]

| **Supplementary File 1: Key Resources Table** | | | | |
| --- | --- | --- | --- | --- |
| **Reagent type (species) or resource** | **Designation** | **Source or reference** | **Identifiers** | **Additional information** |
| cell line (*Homo-sapiens*) | 293T, Epithelial | ATCC | Cat# CRL-3216, RRID:CVCL_0063 | Mycoplasma free |
| cell line (*Homo-sapiens*) | HCT116, Epithelial, *colorectal carcinoma* | ATCC | Cat# CCL-247, RRID:CVCL_0291 | Mycoplasma free |
| cell line (*Homo-sapiens*) | Flp-In  T-Rex 293,  Epithelial | Invitrogen | Cat# R78007,  RRID:CVCL_U427 | Mycoplasma free |
| cell line (*Homo-sapiens*) | 293-FRT-Flag-HA USP21-WT | This paper |  | Doxycycline-inducible, Mycoplasma free,  Established in the Bennett Lab (UCSD) |
| cell line (*Homo-sapiens*) | 293-FRT-Flag-HA USP21-CS | This paper |  | C250S catalytic mutant, Doxycycline-inducible,  Mycoplasma free, Established in the Bennett Lab (UCSD) |
| cell line (*Homo-sapiens*) | 293-FRT-Flag-HA OTUD3-WT | This paper |  | Doxycycline-inducible,  Mycoplasma free, Established in the Bennett Lab (UCSD) |
| cell line (*Homo-sapiens*) | 293-FRT-Flag-HA OTUD3-CS | This paper |  | C76S catalytic mutant,  Doxycycline-inducible,  Mycoplasma free, Established in the Bennett Lab (UCSD) |
| cell line (*Homo-sapiens*) | HCT116-ZNF598-KO | Sundaramoorthy et. al. (2017) | PMID: 28132843 | C29A catalytic mutant,  Mycoplasma free |
| cell line (*Homo-sapiens*) | HCT116-eS10-KI | This paper |  | K138R/K139R mutations,  Mycoplasma free, Established in the Bennett Lab (UCSD) |
| cell line (*Homo-sapiens*) | HCT116-uS10-KI | This paper |  | K4R/K8R mutation,  Mycoplasma free, Established in the Bennett Lab (UCSD) |
| cell line (*Homo-sapiens*) | HCT116-uS3-KI | This paper |  | K214R mutation,  Mycoplasma free, Established in the Bennett Lab (UCSD) |
| cell line (*Homo-sapiens*) | HCT116-uS5-KI | This paper |  | K54R/K58R mutation,  Mycoplasma free, Established in the Bennett Lab (UCSD) |
| cell line (*Homo-sapiens*) | 293T-USP21-KO | This paper |  | Mycoplasma free, Established in the Bennett Lab (UCSD) |
| cell line (*Homo-sapiens*) | 293T-OTUD3-KO | This paper |  | Mycoplasma free, Established in the Bennett Lab (UCSD) |
| cell line (*Homo-sapiens*) | 293T-USP21/  OTUD3-KO | This paper |  | Mycoplasma free, Established in the Bennett Lab (UCSD) |
| Antibody | anti-RPS2 (Rabbit monoclonal) | Bethyl Laboratories | Cat# A303-794A; RRID:AB_11218192 | WB (1:10,000) |
| Antibody | anti-RPS3 (Rabbit monoclonal) | Bethyl Laboratories | Cat# A303-840A; RRID:AB_2615588 | WB (1:10,000) |
| Antibody | anti-RPS10 (Rabbit monoclonal) | Abcam | Cat# ab151550, RRID:AB_2714147 | WB (1:1000) |
| Antibody | anti-RPS10 (Rabbit polyclonal) | ABclonal | Cat# A6056, RRID:AB_2766730 | WB (1:2000) |
| Antibody | anti-RPS20 (Rabbit monoclonal) | Abcam | Cat# ab133776, RRID:AB_2714148 | WB (1:1000) |
| Antibody | anti-ZNF598 (Rabbit polyclonal) | Sigma | Cat# HPA041760; RRID:AB_10792490 | WB (1:1000) |
| Antibody | anti-OTUD3 (Rabbit polyclonal) | Abcam | Cat# ab107646, RRID:AB_10859864 | WB (1:1000) |
| Antibody | anti-USP21 (Rabbit polyclonal) | Abcam | Cat# ab171028, RRID:AB_2827166 | WB (1:500) |
| Antibody | anti-USP21 (Rabbit polyclonal) | Sigma | Cat# HPA028397;  RRID:AB_10603227 | WB (1:1000) |
| Antibody | anti-p-eIF2a (Rabbit monoclonal) | Cell Signalling Technology | Cat# 3398S, RRID:AB_2096481 | WB (1:1000) |
| Antibody | Anti-C-Myc (9E10) (Mouse monoclonal) | Santa Cruz | Cat# sc-40, RRID:AB_627268 | WB (1:1000) |
| Antibody | anti-ubiquitin (Mouse monoclonal) | EMD Millipore/  Chemicon | Cat# MAB1510; RRID:AB_2180556 | WB (1:5000) |
| Antibody | anti-HA (Mouse monoclonal) | Biolegend | Cat# MMS-101P; RRID:AB_2314672 | WB (1:2000) |
| Antibody | anti-tubulin (Mouse monoclonal) | Cell Signalling Technology | Cat# 3873S; RRID:AB_1904178 | WB (1:5000) |
| Antibody | Anti-Rabbit IgG (H+L), HRP Conjugate antibody | Promega | Cat# W4011; RRID:AB_430833 | WB (1:10,000) |
| Antibody | Anti-Mouse IgG (H+L), HRP Conjugate antibody | Promega | Cat# W4021; RRID:AB_430834 | WB (1:10,000) |
| recombinant DNA reagent | CMV-GFP-2A-VHP-2A-CHFP (Plasmid) | Juszkiewicz and Hegde (2017) | PMID: 28065601 | VHP linker control |
| recombinant DNA reagent | CMV-GFP-2A-VHP-K20-2A-CHFP (Plasmid | Sundaramoorthy et. al. (2017) | PMID: 28132843 | K20 stall reporter |
| recombinant DNA reagent | CMV-N-Myc-USP21 (plasmid) | This paper |  | Established in the Bennett Lab (UCSD) |
| recombinant DNA reagent | CMV-N-Myc-USP21CS  (plasmid) | This paper |  | C250S mutation, Established in the Bennett Lab (UCSD) |
| recombinant DNA reagent | CMV-N-Myc-OTUD3  (plasmid) | This paper |  | Established in the Bennett Lab (UCSD) |
| recombinant DNA reagent | CMV-N-Myc-OTUD3CS  (plasmid) | This paper |  | C76S mutation, Established in the Bennett Lab (UCSD) |
| recombinant DNA reagent | CMV-N-Myc-OTUB2  (plasmid) | This paper |  | Established in the Bennett Lab (UCSD) |
| recombinant DNA reagent | CMV-N-Myc-OTUB2CS  (plasmid) | This paper |  | C15S mutation, Established in the Bennett Lab (UCSD) |
| recombinant DNA reagent | CMV-N-Myc-OTUD1  (plasmid) | This paper |  | Established in the Bennett Lab (UCSD) |
| recombinant DNA reagent | CMV-N-Myc-OTUD1CS  (plasmid) | This paper |  | C320S mutation, Established in the Bennett Lab (UCSD) |
| recombinant DNA reagent | CMV-N-Myc-UCHL1  (plasmid) | This paper |  | Established in the Bennett Lab (UCSD) |
| recombinant DNA reagent | CMV-N-Myc-UCHL1CS  (plasmid) | This paper |  | C90S mutation,  Established in the Bennett Lab (UCSD) |
| recombinant DNA reagent | CMV-N-Myc-USP10  (plasmid) | This paper |  | Established in the Bennett Lab (UCSD) |
| recombinant DNA reagent | CMV-N-Myc-USP10CS  (plasmid) | This paper |  | C424S mutation,  Established in the Bennett Lab (UCSD) |
| commercial assay or kit | BCA Protein Assay | Thermo Scientific (Pierce) | Cat# 23225 |  |
| chemical compound, drug | DL-Dithiothreitol (DTT) | ACROS organics | Cat# 165680050 | (5mM) |
| chemical compound, drug | Anisomycin (ANS) | Fisher Scientific | Cat# 50995788 | (5ug/ml) |
| chemical compound, drug | Harringtonine (HTN) | LKT labs | Cat# H0169 | (2ug/ml) |
| chemical compound, drug | Doxycycline hydrochloride | Fisher Scientific | Cat# BP2653-5 | (2ug/ml) |
| chemical compound, drug | Ammonium persulfate (≥ 98%) | Sigma | Cat# A3678 | (10%) |
| chemical compound, drug | Fetal Bovine Serum | VWR | Cat# 97068-085 |  |
| chemical compound, drug | Trypsin | Sigma-Aldrich | Cat# T1426 |  |
| chemical compound, drug | N-Ethylmaleimide | Sigma-Aldrich | Cat# E3876 | (40mM) |
| software, algorithm | FlowJo (v10.4.1) | BD biosciences | RRID:SCR_008520 |  |
| software, algorithm | Prism 7.0 | GraphPad software | RRID:SCR:002798 |  |
| Other | Lipofectamine RNAiMax | Thermo-Fisher | Cat# 13778030 |  |
| Other | Lipofectamine 2000 | THermo-Fisher | Cat# 11668019 |  |
| Other | Mirus TransIT 293 | Mirus Bio llc | Cat# MIR 2700 |  |
| Other | Protease inhibitor cocktail tablet | Roche | Cat# 11836170001 |  |
| Other | Immun-Blot® PVDF Membrane | BioRad | Cat# 1620177 |  |
| Other | Albumin Standard | Thermo Scientific | Cat# 23209 |  |
| Other | Restore^TM^ Western Blot Stripping Buffer | Thermo Scientific | Cat# 21059 |  |
| Other | Clarity^TM^ Western ECL Substrate | BioRad | Cat# 170-5061 |  |
| sequenced-based reagent | siRNA: ALG13 | Dharmacon | D-014459-01 | GCUAUGAUAACUUCUCUUA |
| sequenced-based reagent | siRNA: ALG13 | Dharmacon | D-014459-02 | CCAGAUGAGUUGUGUGAAU |
| sequenced-based reagent | siRNA: ALG13 | Dharmacon | D-014459-04 | CUGGCAAUGUUAUGUCUAA |
| sequenced-based reagent | siRNA: ALG13 | Dharmacon | D-014459-13 | AGUUGAAGACUGCGAUUAA |
| sequenced-based reagent | siRNA: CYLD | Dharmacon | D-004609-01 | CGAAGAGGCUGAAUCAUAA |
| sequenced-based reagent | siRNA: CYLD | Dharmacon | D-004609-02 | GAACAGAUUCCACUCUUUA |
| sequenced-based reagent | siRNA: CYLD | Dharmacon | D-004609-03 | GAACUCACAUGGUCUAGAA |
| sequenced-based reagent | siRNA: CYLD | Dharmacon | D-004609-04 | GGACAUGGAUAACCCUAUU |
| sequenced-based reagent | siRNA: FAM63A | Dharmacon | D-017114-01 | CAAUGACAGUGCUGCCUAA |
| sequenced-based reagent | siRNA: FAM63A | Dharmacon | D-017114-03 | GGGUGAACUUAGCGUCUUU |
| sequenced-based reagent | siRNA: FAM63A | Dharmacon | D-017114-17 | UGGAUGUCAAUGUGCGAUU |
| sequenced-based reagent | siRNA: FAM63A | Dharmacon | D-017114-18 | GGAAGUGAUCACAUCGGAU |
| sequenced-based reagent | siRNA: FAM63B | Dharmacon | D-022254-01 | GAACAGAAUAUGAGUGAUG |
| sequenced-based reagent | siRNA: FAM63B | Dharmacon | D-022254-02 | AUAGUGAGCUGGUUAGUGA |
| sequenced-based reagent | siRNA: FAM63B | Dharmacon | D-022254-03 | GAGAUUACAUGCUUGAUGC |
| sequenced-based reagent | siRNA: FAM63B | Dharmacon | D-022254-04 | CGGGACAAUCUGUGUAUCA |
| sequenced-based reagent | siRNA:  FAM105A | Dharmacon | D-020519-01 | GUAAUUGGAUUCAGCAGUA |
| sequenced-based reagent | siRNA:  FAM105A | Dharmacon | D-020519-02 | CGACAAGUAAGGAGAGAUA |
| sequenced-based reagent | siRNA:  FAM105A | Dharmacon | D-020519-03 | GACUAGAGCAGAUUGAUAU |
| sequenced-based reagent | siRNA:  FAM105A | Dharmacon | D-020519-04 | UCCGGAGGCUACAUUUAUA |
| sequenced-based reagent | siRNA: FAM188A | Dharmacon | D-008465-01 | GAAUGAAACUUCUUGGUAU |
| sequenced-based reagent | siRNA: FAM188A | Dharmacon | D-008465-02 | UAAAUUAGAUCCAGAAGGA |
| sequenced-based reagent | siRNA: FAM188A | Dharmacon | D-008465-03 | UGUCAUACCUUGUGUGAUA |
| sequenced-based reagent | siRNA: FAM188A | Dharmacon | D-008465-04 | UGAGGAAACUGCUAGUAUU |
| sequenced-based reagent | siRNA: FAM188B | Dharmacon | D-025310-01 | CAAAGCAGAUGGAGUCUUA |
| sequenced-based reagent | siRNA: FAM188B | Dharmacon | D-025310-02 | CAACAGAAAUGAUCUUCGA |
| sequenced-based reagent | siRNA: FAM188B | Dharmacon | D-025310-03 | GGAAAUACGGCUAACAAUU |
| sequenced-based reagent | siRNA: FAM188B | Dharmacon | D-025310-04 | GCACAUGGCUACUGUACAC |
| sequenced-based reagent | siRNA: MPND | Dharmacon | D-018630-02 | GGUGAACCCUGCCAAGAAG |
| sequenced-based reagent | siRNA: MPND | Dharmacon | D-018630-17 | UCAACAAGUUCCAGCCGUU |
| sequenced-based reagent | siRNA: MPND | Dharmacon | D-018630-18 | ACGGAAGGAUCAUGUGGCA |
| sequenced-based reagent | siRNA: MPND | Dharmacon | D-018630-19 | UCGACAAGCUUAAGAUCUC |
| sequenced-based reagent | siRNA:  MYSM1 | Dharmacon | D-005905-01 | GAAGAGAACUGUACAAAGG |
| sequenced-based reagent | siRNA:  MYSM1 | Dharmacon | D-005905-03 | CAGAUUACCUGCCUGGUUA |
| sequenced-based reagent | siRNA:  MYSM1 | Dharmacon | D-005905-05 | GCAGUGAUCUCUUGUUAGA |
| sequenced-based reagent | siRNA:  MYSM1 | Dharmacon | D-005905-18 | CAGUAAAGUGGACGAUAGA |
| sequenced-based reagent | siRNA:  OTUB2 | Dharmacon | D-010983-01 | CCGUUUACCUGCUCUAUAA |
| sequenced-based reagent | siRNA:  OTUB2 | Dharmacon | D-010983-02 | GAGCAGACUUCUUCCGGCA |
| sequenced-based reagent | siRNA:  OTUB2 | Dharmacon | D-010983-03 | AGAGAAGGAUGGCUCAGUG |
| sequenced-based reagent | siRNA:  OTUD1 | Dharmacon | D-026487-01 | ACGAGAAGCUGGCCCUAUA |
| sequenced-based reagent | siRNA:  OTUD1 | Dharmacon | D-026487-03 | ACGAAGAACUUGCCAAAUC |
| sequenced-based reagent | siRNA:  OTUD1 | Dharmacon | D-026487-04 | UAUCAUUCGCUGCUGCCCAA |
| sequenced-based reagent | siRNA:  OTUD3 | Dharmacon | D-027582-01 | GAAAUCAGGGCUUAAAUGA |
| sequenced-based reagent | siRNA:  OTUD3 | Dharmacon | D-027582-02 | UCGCAAAGGUCACAAACAA |
| sequenced-based reagent | siRNA:  OTUD3 | Dharmacon | D-027582-03 | UAAUGCAACUGGAUGUUCA |
| sequenced-based reagent | siRNA:  OTUD6A | Dharmacon | D-032033-01 | GAGAAAGAAUGGAGUCCGA |
| sequenced-based reagent | siRNA:  OTUD6A | Dharmacon | D-032033-02 | GCCCAGAUCCGGAGCUUAA |
| sequenced-based reagent | siRNA:  OTUD6A | Dharmacon | D-032033-03 | CAUUGAAUCUGUCGUCGAA |
| sequenced-based reagent | siRNA:  OTUD6A | Dharmacon | D-032033-04 | ACGACGACUUCAUGAUCUA |
| sequenced-based reagent | siRNA:  OTUD7A | Dharmacon | D-008841-02 | UGUCCUAGCCCAUAUAUUA |
| sequenced-based reagent | siRNA:  OTUD7A | Dharmacon | D-008841-03 | GGACGACAUUGCCCAAGAA |
| sequenced-based reagent | siRNA:  OTUD7A | Dharmacon | D-008841-04 | GCACACACUUCAGCAAGAA |
| sequenced-based reagent | siRNA:  OTUD7A | Dharmacon | D-008841-17 | GGGAAAGACGACAACGAUA |
| sequenced-based reagent | siRNA:  OTULIN | Dharmacon | D-018991-01 | ACAGAUAGCUUGUGAUGAA |
| sequenced-based reagent | siRNA:  OTULIN | Dharmacon | D-018991-02 | GGACUGAAAUUUGAUGGGA |
| sequenced-based reagent | siRNA:  OTULIN | Dharmacon | D-018991-03 | GUACAACACGGAAGAAUUC |
| sequenced-based reagent | siRNA:  OTULIN | Dharmacon | D-018991-17 | GGGCAUCAGAACCGAGAUU |
| sequenced-based reagent | siRNA:  USP6 | Dharmacon | D-006096-05 | CAACGGACCUGGAUAUAGG |
| sequenced-based reagent | siRNA:  USP6 | Dharmacon | D-006096-07 | GCGGAGAGGUUCACAACAA |
| sequenced-based reagent | siRNA:  USP6 | Dharmacon | D-006096-08 | GAGCGGAAGGACAUACUUA |
| sequenced-based reagent | siRNA:  USP6 | Dharmacon | D-006096-09 | GAACCUGAUUGACGGGAUC |
| sequenced-based reagent | siRNA:  USP9X | Dharmacon | D-006099-01 | CAAAGGAGAUUUACUAGAA |
| sequenced-based reagent | siRNA:  USP9X | Dharmacon | D-006099-03 | AGAAAUCGCUGGUAUAAAU |
| sequenced-based reagent | siRNA:  USP9X | Dharmacon | D-006099-04 | ACACGAUGCUUUAGAAUUU |
| sequenced-based reagent | siRNA:  USP9X | Dharmacon | D-006099-05 | GUACGACGAUGUAUUCUCA |
| sequenced-based reagent | siRNA:  USP9Y | Dharmacon | D-006100-01 | GGAAUGAAAUGCUUUGAAA |
| sequenced-based reagent | siRNA:  USP9Y | Dharmacon | D-006100-02 | GUACGGCGAUGUAUUGUUA |
| sequenced-based reagent | siRNA:  USP9Y | Dharmacon | D-006100-03 | GAAUGUACCUGCUACCUUU |
| sequenced-based reagent | siRNA:  USP9Y | Dharmacon | D-006100-05 | GCAGUUGUCCUGUUGCUUA |
| sequenced-based reagent | siRNA:  USP21 | Dharmacon | D-006071-01 | GUACAAAGAUUCCCUCGAA |
| sequenced-based reagent | siRNA:  USP21 | Dharmacon | D-006071-02 | GAACCUGAGUUAAGUGAUG |
| sequenced-based reagent | siRNA:  USP21 | Dharmacon | D-006071-03 | GAGCUGUCUUCCAGAAAUA |
| sequenced-based reagent | siRNA:  USP10 | Dharmacon | D-006062-05 | CCAUAAAGAUUGCAGAGUU |
| sequenced-based reagent | siRNA:  USP10 | Dharmacon | D-006062-06 | CAAACAAGAGGUUGAGAUA |
| sequenced-based reagent | siRNA:  USP10 | Dharmacon | D-006062-08 | CCACAUAUAUUUACAGACU |
| sequenced-based reagent | siRNA:  USP24 | Dharmacon | D-006073-03 | GGACGAGAAUUGAUAAAGA |
| sequenced-based reagent | siRNA:  USP24 | Dharmacon | D-006073-05 | AGGGAAACCUUACCUGUUA |
| sequenced-based reagent | siRNA:  USP24 | Dharmacon | D-006073-06 | CCACAGCUUUGUUGAAUGA |
| sequenced-based reagent | siRNA:  USP24 | Dharmacon | D-006073-07 | GUAGAAGCCUUGUUGUUCA |
| sequenced-based reagent | siRNA:  USP27X | Dharmacon | D-031532-02 | UCAUGUGCCCUAUAAGUUA |
| sequenced-based reagent | siRNA:  USP27X | Dharmacon | D-031532-03 | GAUAUGACGCCGUUUAUGG |
| sequenced-based reagent | siRNA:  USP27X | Dharmacon | D-031532-04 | GAUGUGAGAUGCCGAGUCC |
| sequenced-based reagent | siRNA:  USP27X | Dharmacon | D-031532-05 | UAGCAGUAGACCUGUAUUA |
| sequenced-based reagent | siRNA:  USP31 | Dharmacon | D-022513-01 | GAACCAAGCGACAGUCAUA |
| sequenced-based reagent | siRNA:  USP31 | Dharmacon | D-022513-02 | GUAGACAGCUCUCCAGUCA |
| sequenced-based reagent | siRNA:  USP31 | Dharmacon | D-022513-03 | CCUCAAACCUGCACUUUAU |
| sequenced-based reagent | siRNA:  USP31 | Dharmacon | D-022513-04 | CAGCAUACAUCCUCUUCUA |
| sequenced-based reagent | siRNA:  USP34 | Dharmacon | D-006082-01 | GAAAUUGACUCUCCUUAUU |
| sequenced-based reagent | siRNA:  USP34 | Dharmacon | D-006082-02 | UAACAUGGCUGACUUAAUG |
| sequenced-based reagent | siRNA:  USP34 | Dharmacon | D-006082-03 | GCAAUGAGGUUAAUUCUAG |
| sequenced-based reagent | siRNA:  USP34 | Dharmacon | D-006082-04 | GGACCAAAUUUACAUAUUG |
| sequenced-based reagent | siRNA:  USP35 | Dharmacon | D-006083-02 | AGAGCGAGCUGGCGGGUUU |
| sequenced-based reagent | siRNA:  USP35 | Dharmacon | D-006083-03 | GCUCGGAGUAUCUGAAGUA |
| sequenced-based reagent | siRNA:  USP35 | Dharmacon | D-006083-04 | CAACAUCCUUUACCUACAG |
| sequenced-based reagent | siRNA:  USP35 | Dharmacon | D-006083-05 | GGGCUUUGAUGAAGACAAG |
| sequenced-based reagent | siRNA:  USP40 | Dharmacon | D-006088-01 | GAAACUAGCUGUUAUACAU |
| sequenced-based reagent | siRNA:  USP40 | Dharmacon | D-006088-02 | GAAGAGAAAUGGGUCACUA |
| sequenced-based reagent | siRNA:  USP40 | Dharmacon | D-006088-03 | GCAGAGAGUUGCCGAUUUC |
| sequenced-based reagent | siRNA:  USP40 | Dharmacon | D-006088-04 | GAACGAGCCUGCGCAAGUU |
| sequenced-based reagent | siRNA:  USP41 | Dharmacon | D-031434-03 | GGAAGAAGACCCGUGGGAA |
| sequenced-based reagent | siRNA:  USP41 | Dharmacon | D-031434-04 | CCAGGGAGUUAUCAAGCAA |
| sequenced-based reagent | siRNA:  USP41 | Dharmacon | D-031434-17 | GGAAUUCACAGACGAGAAA |
| sequenced-based reagent | siRNA:  USP41 | Dharmacon | D-031434-18 | GCGAGAGUCUUGUGAUGCU |
| sequenced-based reagent | siRNA:  USP47 | Dharmacon | D-006093-01 | GGACUUGACUCUCACAGUA |
| sequenced-based reagent | siRNA:  USP47 | Dharmacon | D-006093-02 | CCUGAAAGCUGAAGGAUUU |
| sequenced-based reagent | siRNA:  USP47 | Dharmacon | D-006093-03 | GAGAGAAGCUUAGUGAAAU |
| sequenced-based reagent | siRNA:  USP47 | Dharmacon | D-006093-04 | GCAACGAUUUCUCCAAUGA |
| sequenced-based reagent | siRNA:  USP51 | Dharmacon | D-032247-01 | GAGCAGGGAUGACCACAUA |
| sequenced-based reagent | siRNA:  USP51 | Dharmacon | D-032247-02 | AAACAAAGCAGCACCAUUU |
| sequenced-based reagent | siRNA:  USP51 | Dharmacon | D-032247-04 | GCUUUAAAGUAGGUAAGAA |
| sequenced-based reagent | siRNA:  USP51 | Dharmacon | D-032247-17 | GGUUGAUCUACCAGCGUUU |
| sequenced-based reagent | siRNA:  ZNF598 | Dharmacon | D-007104-01 | GCACCAAGUCCAAGAAGAA |
| sequenced-based reagent | siRNA:  ZNF598 | Dharmacon | D-007104-03 | GAAAGGUGUACGCAUUGUA |
| sequenced-based reagent | siRNA:  ZNF598 | Dharmacon | D-007104-04 | ACGAGGAGGUGGACAGGUA |
| sequenced-based reagent | siRNA:  Non-Targeting Pool | Dharmacon | D-001206-14-05 | UAAGGCUAUGAAGAGAUAC, AUGUAUUGGCCUGUAUUAG, AUGAACGUGAAUUGCUCAA, UGGUUUACAUGUCGACUAA |
